# The serine protease matriptase inhibits migration and proliferation in multiple myeloma cells

**DOI:** 10.18632/oncotarget.28300

**Published:** 2022-10-20

**Authors:** Ida Steiro, Esten N. Vandsemb, Samah Elsaadi, Kristine Misund, Anne-Marit Sponaas, Magne Børset, Pegah Abdollahi, Tobias S. Slørdahl

**Affiliations:** ^1^Center for Myeloma Research, Department of Clinical and Molecular Medicine, Faculty of Medicine and Health Sciences, Norwegian University of Science and Technology (NTNU), Trondheim, Norway; ^2^Department of Immunology and Transfusion Medicine, St. Olav's University Hospital, Trondheim, Norway; ^3^Laboratory Clinic, St. Olav's University Hospital, Trondheim, Norway; ^4^Department of Hematology, St. Olav's University Hospital, Trondheim, Norway

**Keywords:** matriptase, multiple myeloma, tumor suppressor, migration, Src

## Abstract

Background: Multiple myeloma (MM) is an incurable malignancy of plasma cells. The serine protease matriptase is frequently dysregulated in human carcinomas, which facilitates tumor progression and metastatic dissemination. The importance of matriptase in hematological malignancies is yet to be clarified. In this study, we aimed to characterize the role of matriptase in MM.

Materials and Methods: mRNA expression of matriptase and its inhibitors hepatocyte growth factor activator inhibitor (HAI)-1 and HAI-2 was studied in primary MM cells from patient samples and human myeloma cell lines (HMCLs). We further investigated the effect of matriptase on migration and proliferation of myeloma cells *in vitro*. By use of the CoMMpass database, we assessed the clinical relevance of matriptase in MM patients.

Results: Matriptase was expressed in 96% of patient samples and all HMCLs tested. Overexpression of matriptase *in vitro* reduced proliferation, and significantly decreased cytokine-induced migration. Conversely, matriptase knockdown significantly enhanced migration. Mechanistically, overexpression of matriptase inhibited activation of Src kinase.

Conclusions: Our findings may suggest a novel role of matriptase as a tumor suppressor in MM pathogenesis.

## INTRODUCTION

Multiple myeloma (MM) is characterized by a clonal expansion of bone marrow (BM) plasma cells. The interaction between the myeloma cells and the BM microenvironment plays a crucial role in homing to the BM marrow, proliferation and survival of the cancer cells, and in drug resistance [1–3]. Over the last two decades survival rates for myeloma patients have significantly improved by the introduction of novel therapies such as immunomodulatory drugs, proteasome inhibitors and monoclonal antibodies [4]. However, MM remains incurable due to the inevitable course of relapse and development of refractory disease.

Matriptase (*ST14*), a type-II transmembrane serine protease primarily found in epithelial tissues, is overexpressed in a variety of human malignancies [5, 6]. In mice, modest overexpression of matriptase caused malignant transformation of the epidermis [7]. Under physiological conditions, matriptase’s proteolytic activity is tightly controlled by its cognate inhibitor hepatocyte growth factor activator inhibitor (HAI)-1 (*SPINT1*), which paradoxically also is required for proper expression and zymogen activation of matriptase [8–10]. These opposing roles also apply to HAI-2 (*SPINT2*), another membrane-anchored Kunitz-type serine protease inhibitor closely related to HAI-1 [11–14]. However, the exact role of HAI-2 in the life cycle of matriptase remains to be clarified. Dysregulated expression of matriptase and its inhibitors causing an increase in matriptase´s enzymatic activity has been associated with cancer growth, survival, and metastasis [15–17]. These events have been linked to the proteolytic activation of growth factors such as hepatocyte growth factor (HGF) and macrophage-stimulating protein 1 (MSP-1), and processing of downstream protease systems including urokinase-type plasminogen activator (uPA) [17, 18].

In carcinomas, expression levels of matriptase are comparable with those found in the normal epithelia from which the tumor cells originate [19]. Additionally, the majority of matriptase-expressing epithelial cancers co-express both HAI-1 and HAI-2 [20]. Among hematological malignancies, high matriptase expression is reported in non-Hodgkin B-cell lymphoma, chronic lymphocytic leukemia, and acute myeloid leukemia [21–23]. However, in contrast to epithelial cancers, many neoplastic B-cells express matriptase in the absence of or with only minor levels of HAI-1 [21, 22]. In normal B-lymphocytes, the expression of matriptase is still to be clarified [21, 22, 24].

Growth and expansion of MM cells occur almost exclusively within the BM. Dissemination of myeloma cells throughout the BM, a hallmark of MM, is caused by myeloma cells homing to new sites [25]. Several cytokines in the BM microenvironment facilitate homing of myeloma cells, including stromal cell-derived factor (SDF)-1α and hepatocyte growth factor (HGF), both of which have been shown to mediate the activation of Src kinase [26–29]. As the prototype member of the Src family kinases (SFK), the effects of increased Src activity in carcinogenesis have been extensively studied [30, 31]. Among others, it has been established as a critical player in metastasis formation promoting cancer cell motility and invasiveness [30].

Previous research on matriptase in carcinogenesis have almost exclusively focused on epithelial cancers, while the knowledge about this serine protease in hematological malignancies remains scarce. In this study, we investigated the functional role of matriptase *in vitro* using human multiple myeloma cells as a model system. We also explored the clinical relevance of matriptase expression using the publicly available MMRF CoMMpass dataset. Our findings may indicate a novel role of matriptase in MM pathogenesis.

## RESULTS

### Matriptase is associated with myeloma cell proliferation

**Figure 1:**
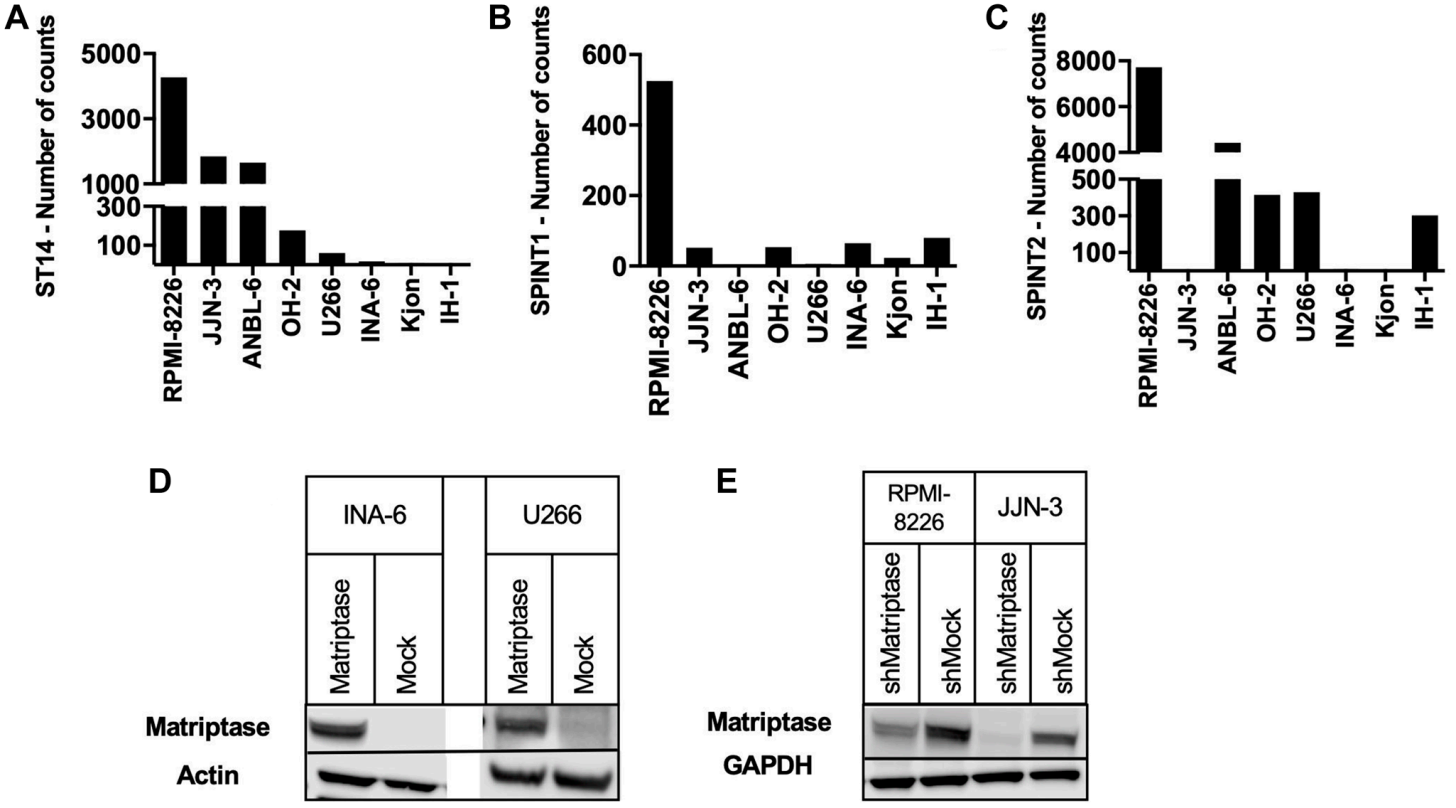
Matriptase, HAI-1 and HAI-2 expression in human myeloma cell lines. (**A**–**C**) Nanostring mRNA expression screening of (A) matriptase (ST14), (B) HAI-1 (SPINT1) and (C) HAI-2 (SPINT2) was performed on human myeloma cell lines (*n* = 8). (**D**, **E**) Matriptase (D) overexpression and (E) knockdown was confirmed via Western blotting.

Initially, we screened for mRNA expression of matriptase, HAI-1 and HAI-2 in eight human myeloma cell lines (Figure 1A–1C). Matriptase (*ST14*) mRNA expression was detected in all but the IH-1 cell line. The levels varied substantially but were overall higher than the mRNA expression levels of HAI-1 and HAI- 2. Based on the screening data, we generated cell lines with overexpression or knockdown of matriptase. In the MM cell line INA-6, with negligible endogenous *ST14* expression, we overexpressed matriptase (INA- 6 Matriptase) and empty control vector (INA-6 Mock). In addition, we performed overexpression studies with transient transfection in the MM cell line U266 (U266 Matriptase and U266 Mock), which also displayed low levels of endogenous *ST14*. Further, we established stable matriptase knockdown in two MM cell lines with high matriptase gene expression; RPMI-8226 (RPMI-8226 shMatriptase) and JJN-3 (JJN-3 shMatriptase), and respective control cells (RPMI-8226 shMock and JJN-3 shMock). Both overexpression and knockdown of matriptase was confirmed by Western blotting (Figure 1D, 1E).

**Figure 2:**
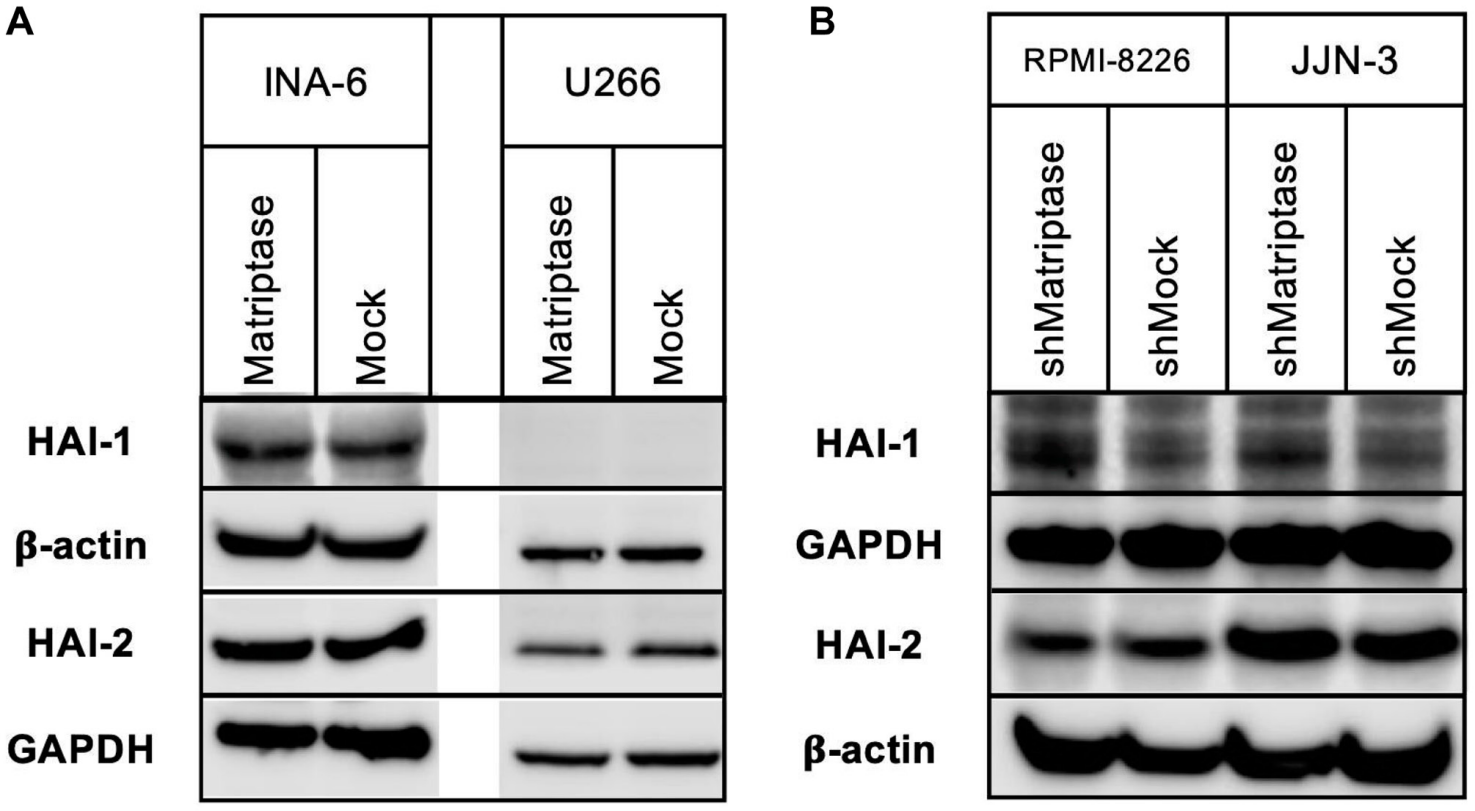
HAI-1 and HAI-2 protein expression in matriptase overexpression and knockdown myeloma cell lines. Protein expression of HAI-1 and HAI-2 was determined using Western blotting. (**A**) INA-6 and U266 matriptase overexpression (Matriptase) and control (Mock) cells, and (**B**) RPMI-8226 and JJN-3 matriptase knockdown (shMatriptase) and control (shMock) cells. One representative of at least three independent experiments is shown.

In normal epithelia, matriptase and the HAIs are ubiquitously co-expressed, and the ratio between them is strictly regulated [32]. The forced alterations of matriptase gene expression in our model could potentially induce changes in the expression of HAI-1 and HAI-2. We therefore investigated expression of HAI-1 and HAI-2 in all four cell lines. At the protein level, no significant changes were observed in either of the cell lines examined, neither of the HAI-1 nor HAI-2 protein (Figure 2).

**Figure 3:**
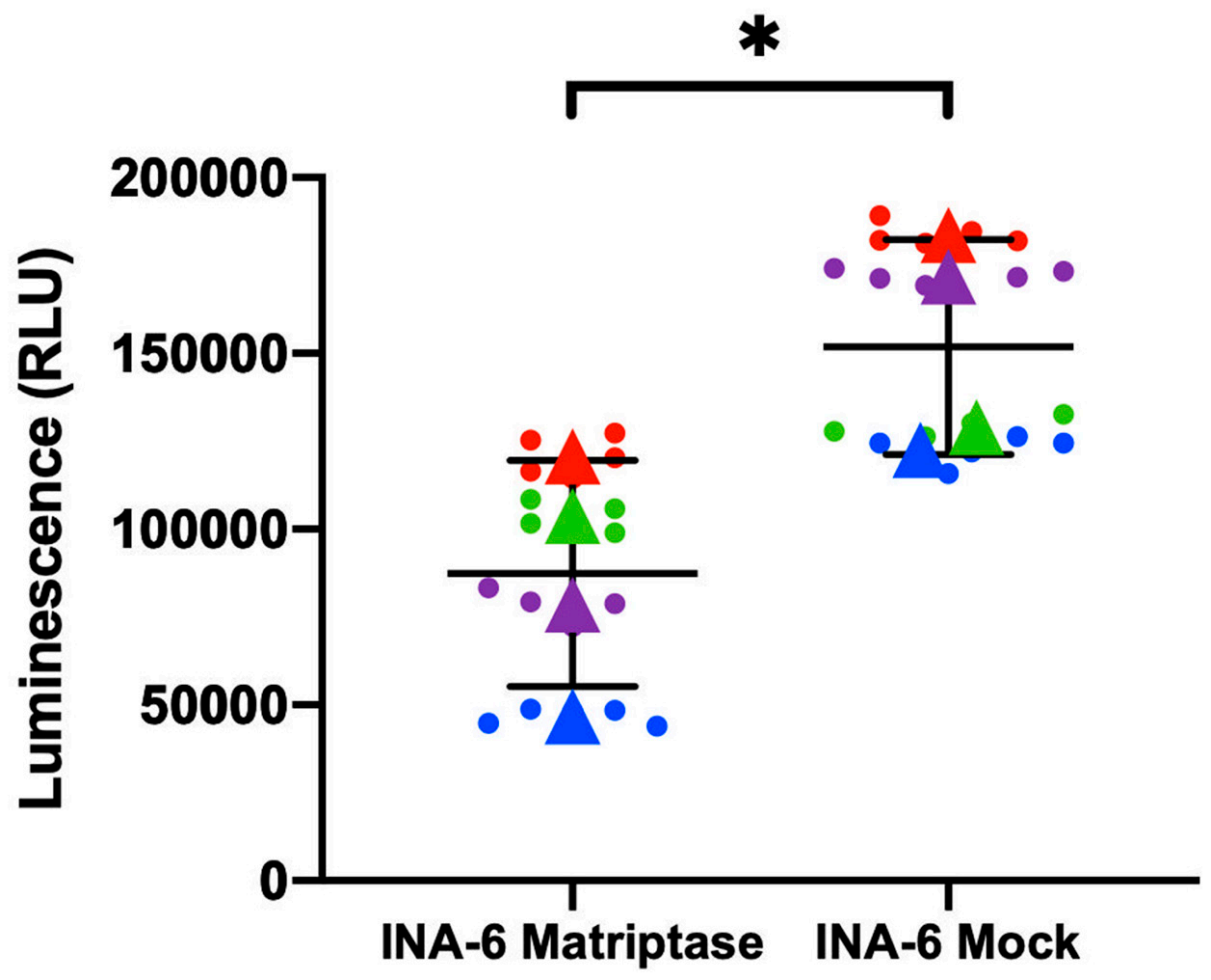
Matriptase overexpression is associated with myeloma cell proliferation. Cell proliferation was measured in INA-6 Matriptase overexpression (Matriptase) and control (Mock) cells by the CellTiter-Glo assay. The mean (±SD) of four independent experiments is shown. Each dot represents one technical replicate and dots in the same color correspond to one of the biological replicates. Triangles represent the mean of each biological replicate. *p*-value was calculated by unpaired Student’s *t*-test based on the average from each independent experiment. ^*^ *p* ≤ 0.05.

Matriptase has been implicated in the survival of cancer cells. To explore the role of matriptase in MM, we investigated the effect of matriptase overexpression and knockdown on MM cell proliferation using the CellTiter-Glo assay. In INA-6 overexpressing matriptase the relative proliferative rate was clearly decreased compared to control cells in repeated experiments (Figure 3). U266 cells with matriptase overexpression displayed the same trend, although the difference did not reach statistical significance (Supplementary Figure 1). However, in RPMI-8226 and JJN-3 MM cell lines with matriptase knockdown, no significant impact on cell proliferation was observed (Supplementary Figure 2). The effect on proliferation in the overexpression cells were not due to reduced viability as assessed by staining with annexin V-PI (Supplementary Figure 3).

### Matriptase inhibited migration of multiple myeloma cell lines

**Figure 4:**
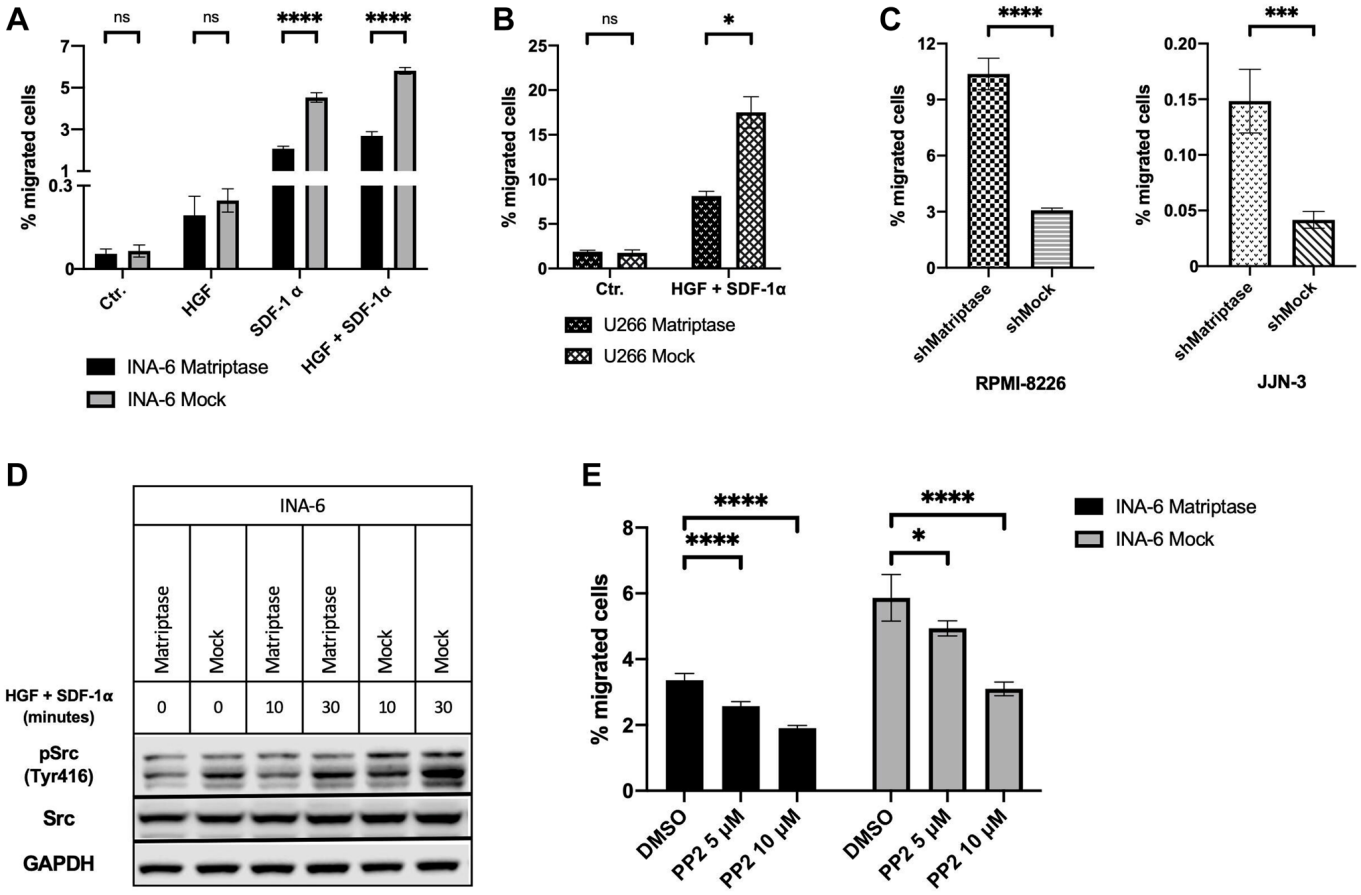
Matriptase inhibits migration in human myeloma cell lines. (**A**, **B**) Migration of (A) INA-6 and (B) U266 matriptase overexpression (Matriptase) and control (Mock) cells. The promigratory cytokines HGF and/or SDF-1α were added to the lower wells as indicated. (**C**) Basal migration of matriptase knockdown (shMatriptase) and control cells (shMock) in RPMI-8226 and JJN-3. (**D**) Src activation in INA-6 Matriptase and Mock. Cells were starved for 6 h in serum-free medium. Subsequently, cells were stimulated with HGF and SDF-1α for 10 and 30 min and probed with antibodies as indicated. (**E**) Migration of INA-6 Matriptase and Mock cells. The combination of HGF and SDF-1α were added to the lower wells in all conditions. DMSO or PP2 Src inhibitor were added in concentrations as indicated to both upper and lower wells. (A–C and E) Cells were seeded in the upper well of a two-chamber transwell migration assay. After 24 h incubation, cells in the bottom wells were counted and the percentage of migrated cells calculated. Bars in (A), (B), (C) and (E) represent the mean (±SD) of at least two repeated counts in two independent measurements. Concentrations of HGF and SDF-1α was 150 ng/mL and 75 ng/mL, respectively, in all experiments. One representative of three independent experiments is shown in all figures. *p*-values were calculated by unpaired Student’s *t*-test. ns = not significant (*p* > 0.05), ^*^ *p* ≤ 0.05, ^***^ *p* ≤ 0.001, ^****^ *p* ≤ 0.0001.

To further examine a possible role of matriptase in MM, we studied the migratory capacity of the MM cells with manipulated matriptase expression. In agreement with previous studies from our group, SDF-1α and/or HGF enhanced the migratory capacity of INA-6 MM cells (Figure 4A) [26, 33]. Matriptase overexpression in INA-6 decreased SDF-1α-induced migration by 54% compared to control cells. Likewise, upon stimulation with the combination of SDF-1α and HGF, a reduction of more than 50% in the number of migrating cells was found in INA-6 Matriptase compared to INA-6 Mock. The same pattern was observed in response to HGF stimulation. Similar significant results were obtained with U266 Matriptase and Mock cells when exposed to the described promigratory cytokine combination (Figure 4B).

Next, we studied migration in RPMI-8226 and JJN-3 matriptase knockdown and control cells. We found that matriptase knockdown significantly enhanced migration compared to control cells (Figure 4C). The difference was present in the unstimulated control condition, where only serum was added the experimental media in the upper and lower compartment. No additional effect was observed in response to cytokine stimulation (Supplementary Figure 4).

### Matriptase blocked cytokine-stimulated migration through inhibition of Src kinase

To identify the mechanism responsible for the difference in migratory capacity after cytokine stimulation, we investigated intracellular signaling pathways. INA- 6 cells overexpressing matriptase and control cells were starved for 6 hours in serum-free medium prior to stimulation with HGF and/or SDF-1α for 10 and 30 minutes. In INA-6 Mock cells, we found a clear link between the degree of cytokine-induced migration and Tyr416 phosphorylation of Src kinase. Src activation was most potent after stimulation with both HGF and SDF-1α (Figure 4D), corresponding to the condition that induced the utmost cell migration. No correlation was observed between activation of PAK1/2, p38 MAPK, p42/44 MAPK or Akt and the degree of cytokine-induced migration in INA-6 cells (Supplementary Figure 5). In RPMI-8226 and JJN-3 cells where matriptase was knocked-down, no difference in Src activation was observed between the knockdown and control cells (Supplementary Figure 6).

### Matriptase overexpression reduced sensitivity to Src inhibition

Further, we examined whether INA-6 matriptase overexpression and control cells were sensitive to Src inhibition. Increasing concentrations of the Src inhibitor PP2 significantly decreased cytokine-induced migration in both matriptase-overexpressing and control cells in a dose-dependent manner (Figure 4E). The reduction in migration was not caused by effects on cell proliferation as determined by the CellTiter-Glo assay (Supplementary Figure 7).

### Matriptase is expressed in MM primary cells with its cognate inhibitors and high expression displays a survival advantage

**Figure 5:**
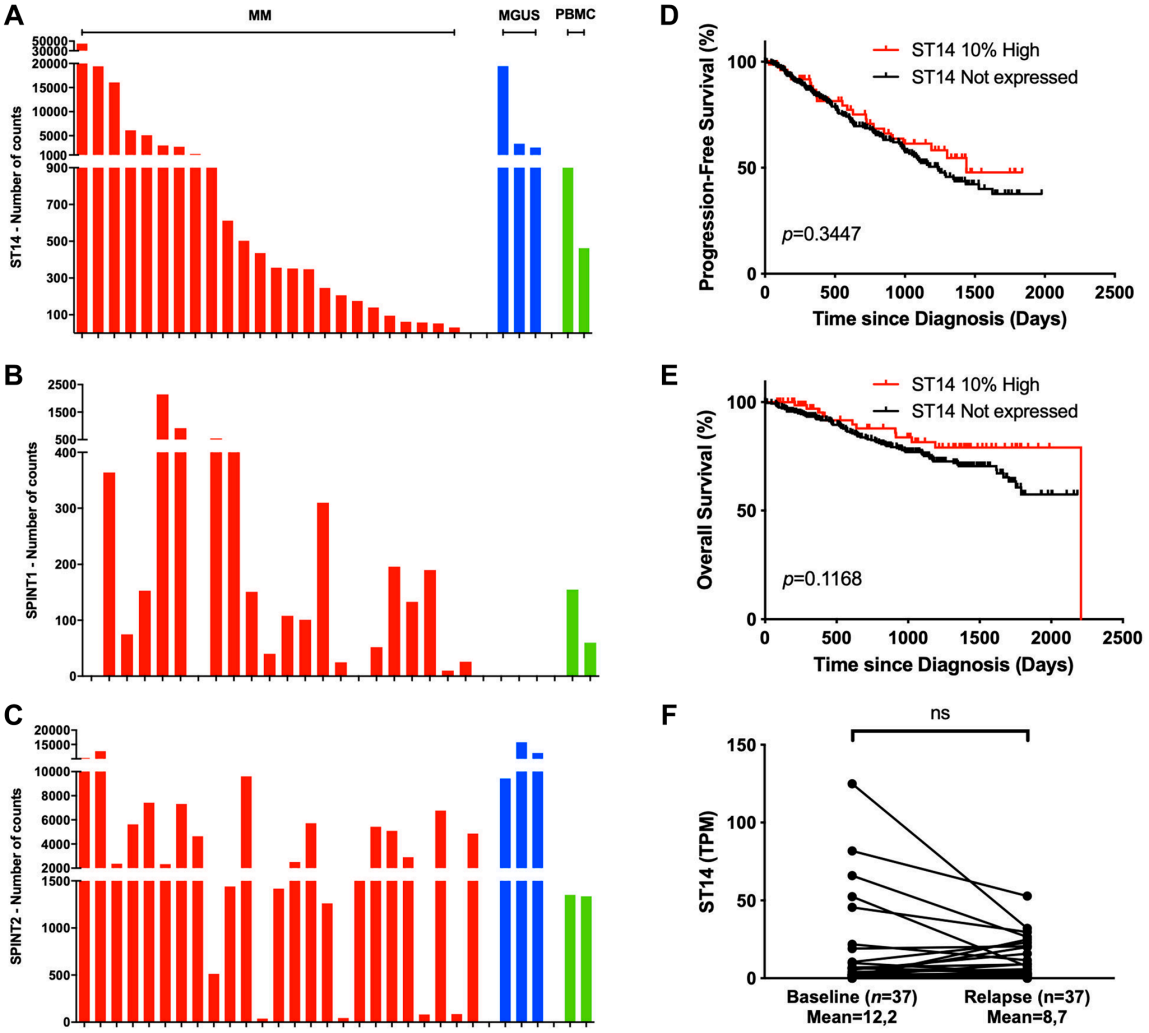
Matriptase is expressed in patient samples and is associated with myeloma cell survival. (**A**–**C**) Nanostring mRNA expression analysis of (A) ST14, (B) SPINT1 and (C) SPINT2 was investigated in primary cells from patients with multiple myeloma (MM, *n* = 25, red) and monoclonal gammopathy of undetermined significance (MGUS, *n* = 3, dark blue), and in peripheral blood mononuclear cells (PBMCs, *n* = 2, green). (**D**, **E**) Kaplan-Meier analysis with log-rank test for (D) progression-free and (E) overall survival data from CoMMpass IA14 cases stratified into the upper 10th percentile (10% high, *n* = 75) and non-expressers (TPM<1.0, *n* = 281). (**F**) ST14 expression at diagnosis and last relapse in RNA-sequenced longitudinal CD138^+^ patient samples from CoMMpass IA14. Significance was determined by Wilcoxon signed-rank test. Abbreviation: ns: not significant. (*p* > 0.05).

Our preliminary screening of the matriptase mRNA in HMCLs revealed high expression levels. To investigate the clinical relevance of our *in vitro* data, we further examined mRNA expression of matriptase and its cognate inhibitors HAI-1 and HAI-2 in 25 MM and three MGUS primary cell samples, and in peripheral blood mononuclear cells (PBMCs) from two healthy donors (Figure 5A–5C). The analysis was performed with Nanostring nCounter mRNA expression profiling. Matriptase (*ST14*) mRNA expression was detected in all samples except for one MM primary cell sample. The expression level was highly variable between samples. HAI-1 (*SPINT1*) mRNA expression was found in 20/25 primary MM cell samples (80%) and both PBMC samples, while absent in plasma cells from MGUS patients. The gene encoding HAI-2 (*SPINT2*) was expressed in primary cells from all MM and MGUS patients, and in both PBMC samples. Among the primary MM cell samples, the ratio of matriptase to HAI-1 was higher than the ratio of matriptase to HAI-2, although not significant (Supplementary Figure 8). Available clinical data of the included MM patients and MGUS patients did not reveal any patterns of significance correlated to the mRNA expression values (Supplementary Table 1).

To determine the clinical relevance of matriptase in MM, we analyzed the prognostic value of *ST14* expression on patient survival data in the publicly available MMRF CoMMpass IA14 dataset. *ST14* was highly expressed in a subset of MM patients (TPM >30, *n* = 79). When patients were grouped into *ST14* high-expressers (upper 10th percentile) and non-expressers a trend towards poor progression-free survival and overall survival was found in the latter group (Figure 5D and 5E). Survival analysis of HAI-1 and HAI-2 gene expression did not reveal any differences (Supplementary Figure 9). Further, we examined *ST14* expression at time of diagnosis and last progression in 37 patients with longitudinal samples from the CoMMpass IA14 dataset. We found that matriptase level was lower at relapse in a proportion of patients (Figure 5F). Although the mean matriptase level was lower at relapse, this difference did not reach statistical significance.

## DISCUSSION

With the current study, we aimed to explore roles of matriptase in the pathogenesis of MM. We demonstrate that matriptase overexpression *in vitro* was associated with reduced myeloma cell proliferation and that matriptase significantly inhibited myeloma cell migration. We show that matriptase mRNA expression was detected at high levels in MM patient samples. Analysis of myeloma patients with high matriptase gene expression levels displayed no significant survival benefit.

Overexpression of matriptase in INA-6 and U266 cells decreased proliferation compared to control cells. Furthermore, overexpression of matriptase in these cells significantly inhibited cytokine-induced migration. The reverse migratory finding in RPMI-8226 and JJN- 3 matriptase knockdown cells, where migration was facilitated compared to control cells, suggests a role of matriptase as a negative regulator of MM cell motility. Contrary to our findings, high expression of matriptase in epithelial carcinomas has been reported to promote motility and invasion, including studies on endometrial, ovarian, and prostate cancer [16, 34, 35]. In a model of Burkitt’s lymphoma, matriptase inhibition reduced cancer cell invasion, but did not have any impact on cell migration or proliferation [22]. Except from these observations, no other study to date has investigated the effect of matriptase on migration of myeloma cells. These contradicting results may be associated with variation in expression of the HAIs, availability, and processing of matriptase substrates, or other unknown mechanisms.

Migration of INA-6 cells was largely induced by SDF-1α. We therefore investigated signaling pathways downstream SDF-1α and its receptor CXC chemokine receptor 4 (CXCR4). Our results demonstrate that matriptase overexpression decreased migration by inhibiting phosphorylation of Src on Tyr416. To our knowledge, no prior studies have described a function of matriptase in Src activation. Src is previously identified as an important molecule in MM pathogenesis with a role in proliferation, survival, and drug resistance [36, 37]. Several studies confirm activation of SFK downstream of the SDF-1α/CXCR4 axis and its involvement in cell motility, consistent with our observations [27, 38]. Investigation of CXCR4 expression by flow cytometry in INA-6 cells revealed high levels in both matriptase overexpressing and control cells (Supplementary Figure 10). Indeed, matriptase has numerous substrates, which possibly could interfere with the SDF-1α/CXCR4 signaling axis [17, 39, 40]. Further research is needed to elucidate these underlying mechanisms.

In contrast to the inhibitory effect on proliferation of matriptase overexpression most abundantly seen in INA-6 cells, the opposite was not observed after knockdown of matriptase in RPMI-8226 and JJN-3 cells. Also, in these cell lines, we were not able to show a difference in Src activation compared to control cells, which may be linked to these cells’ lack of response to cytokine stimulation. An explanation for the lack of consistency between the overexpression and knockdown models may rely on a cell-specific variation in the gene signature caused by altered matriptase expression. Also, as matriptase was knocked down, but not completely knocked out, it might be that the residual *ST14* expression in our cell lines was sufficient to equalize the differences that otherwise would have been detectable. Contrary to the results in the overexpressing cells indicating an inhibitory effect of matriptase on MM cell proliferation, others have found that knockdown of matriptase in human keratinocytes and in a breast cancer mouse model decreased proliferation [41, 42]. The epithelial origin of the two latter models may explain the discrepancy between these and our MM models.

mRNA expression of matriptase, HAI-1 and HAI-2 was detected in the majority of examined patient samples. HAI-1 mRNA expression was not detected in 20% of samples, while HAI-2 mRNA expression was present in all samples – and with an overall higher expression level compared to HAI-1. These observations are in accordance with a recent study on hematological cancer cells, including samples from myeloma patients, which found higher expression levels of HAI-2 than of HAI-1. Our data thus confirm the discrepancy between matriptase-expressing hematological and epithelial cancer cells, in which the latter group, almost without exception, co-express both HAI-1 and HAI-2 [20]. This suggests that matriptase may be differentially regulated in hematological malignancies and epithelial carcinomas. Moreover, our analysis revealed matriptase mRNA expression in both PBMCs and all three MGUS patients. To date, the biological role of such expression remains unclear. In monocytes, matriptase may be involved in regulating plasminogen activation [24].

Our findings of reduced myeloma cell migration and proliferation could suggest a favorable role of matriptase in MM. Thus, we performed survival analyses utilizing the MMRF CoMMpass IA14 dataset to study the clinical relevance of matriptase expression in MM. We did not find any significant correlation to increased OS or PFS in patients with high levels of matriptase gene expression. The decrease in matriptase expression levels between time of diagnosis and progression was also not significant. A previous study on ovarian cancer found a positive correlation between matriptase expression, early clinical stage, and increased survival [43]. Another study have demonstrated downregulation of matriptase in human colon adenomas and adenocarcinomas compared to normal tissue [44]. However, in prostate and bladder cancer, opposite results have been reported [45, 46]. Further research might resolve these inconsistencies and elaborate on the relation between matriptase and the HAIs in MM.

## MATERIALS AND METHODS

### Cell lines and culture conditions

We used the human myeloma cell lines INA-6 (a gift from Dr. M. Gramazki, University of Erlangen-Nuremberg, Erlangen, Germany), JJN-3 (a gift from Dr. I.M. Franklin, University of Birmingham, Birmingham, UK), RPMI-8226 and U266 (both from ATCC, Rockville, MD, USA). The MM cell lines were cultured in RPMI-1640 supplemented with 2 mM l-glutamine (hence referred to as RPMI). INA-6 and JJN-3 were supplemented with 10% heat-inactivated fetal calf serum (FCS), RPMI-8226 with 20% FCS and U266 with 15% FCS. INA-6 is interleukin (IL)-6 dependent and was maintained in media containing 1 ng/mL IL-6. Growth media were replenished twice weekly. Cells were cultured at 37°C in a humidified atmosphere with 5% CO_2_. New stock batches of the cell lines used were thawed at least every 4 months and were regularly tested to ensure the absence of mycoplasma.

### Primary cells

Patient myeloma cells (CD138^+^) were isolated from bone marrow specimens using RoboSep automated cell separator and Human CD138 Positive Selection Kit (StemCell Technologies, Grenoble, France). More than 90% of separated cells were myeloma cells. Peripheral blood mononuclear cells (PBMCs) were isolated from buffy coats of healthy blood donors (blood bank of St. Olavs Hospital, Trondheim, Norway) using gradient centrifugation with Lymphoprep (Axis-Shield, Oslo, Norway). Patient samples were obtained from the Norwegian Myeloma Biobank.

### Antibodies, cytokines, and other reagents

IL-6 was obtained from Invitrogen (Camarillo, CA, USA). SDF-1α was obtained from Peprotech (London, UK) and HGF was obtained from R&D Systems (Abingdon, UK). The antibody against matriptase (D-7, sc-365482) was from Santa Cruz Biotechnology (Santa Cruz, CA, USA), the antibody against HAI-1 (9B10) was obtained from eBioscience (San Diego, CA, USA), and the antibodies against GAPDH (ab8245) and HAI-2 (ab128926) were purchased from Abcam (Cambridge, UK). Antibodies against phosphorylated Src (Tyr416; #6943), PAK1 (Ser144)/PAK2 (Ser141) (#2606), p44/42 MAPK (Erk1/2) (Thr202/Tyr204; #4370) and Akt (Ser473; #9271), total Src (#2123), p44/42 MAPK (Erk1/2) (#4695), Akt (#9272), p38 MAPK (#9212) and β-actin (#4970) were obtained from Cell Signaling Technology (Beverly, MA, USA). Cytokines were diluted to final concentrations in RPMI. Experiments with INA-6 and U266 cells were performed with RPMI supplemented with 2% heat-inactivated human serum (Department of Immunology and Transfusion Medicine, St. Olavs Hospital, Trondheim, Norway) and 0,1 ng/mL IL-6. For starvation purposes cells were incubated with RPMI supplemented with 0,1% bovine serum albumin (BSA; Sigma Aldrich, St. Louis, MO, USA). Experiments with RPMI-8226 and JJN-3 were performed in their respective culture media as previously described. All cell lines were washed in Hanks’ balanced salt solution (HBSS; Sigma Aldrich).

### Lentiviral transduction for matriptase overexpression

For stable matriptase overexpression 293T packaging cells were transduced with pLenti-ST14 or pLenti (control plasmid) in combination with psPAX2 (packaging plasmids) and pMD2.G (envelope plasmid) for virus production. INA-6 cells were transduced with viruses produced by packaging cells to establish INA-6 overexpressing matriptase (INA-6 Matriptase) and control cell line (INA-6 Mock). The pLenti-ST14 was made by performing a LR recombination reaction between the ORF *ST14* cDNA clone: ORFEXPRESS Gateway PLUS shuttle clone (U1384, GeneCopoeia, Rockville, MD, USA) and the pLenti CMV Puro DEST (w118-1). The plasmid was a gift from Eric Campeau & Paul Kaufman (Addgene plasmid #17452) [47]. Transduced cells were grown in medium containing 0.2 μg/mL puromycin for selection. Overexpression was confirmed by Western blotting.

### Transient overexpression

Cells were grown with low density prior to transient overexpression. In total, 5 × 10^6^ cells were pelleted, and the pellet was resuspended in transfection buffer [Amaxa^®^ Cell Line Nucleofector^®^ Kit R (Lonza, Basel, Switzerland)]. Cells were added to separate nucleofection cuvettes containing 2 μg of either pLenti CMV Puro DEST-ST14 or pLenti CMV Puro DEST and transfected by a Nucleofector™ II device (Lonza). Program U-001 was used for U266. Western blot was used to confirm overexpression after 48 h.

### Lentiviral transduction for matriptase knockdown

A plasmid containing a shRNA specific for matriptase (sc-43911-V) and control plasmid (sc-108080) purchased from Santa Cruz Biotechnology was used to establish cells with stable knocked-down matriptase (RPMI-8226 shMatriptase and JJN-3 shMatriptase) and control cell lines (RPMI-8226 shMock and JJN-3 shMock), respectively. Transduced cells were grown in medium containing 0.2 μg/mL puromycin for selection. Knockdown was confirmed by Western blotting.

### Migration assay

Cells were washed three times in HBSS and resuspended in experiment media as previously described. A total of 4 × 10^5^ cells were seeded in 100 μL medium in the upper compartments of polycarbonate Transwell two-chamber migration plates (pore size: 5 μm; Costar, Corning, NY, USA). In experiments ran with INA-6 cells, 150 ng/mL HGF and/or 75 ng/mL SDF-1α was added in the lower chambers. The total volume of medium in the lower compartments was 600 μL. Experiments were run at 37°C in 5% CO_2_. After 24 h, the number of cells that had migrated through the membrane into the lower chamber was determined by a Coulter Counter Z1 (Beckman Coulter, Fullerton, CA, USA).

### CellTiter-glo luminescent cell viability assay

CellTiter-Glo Luminescent (CTG) Cell Viability Assay (Promega, Madison, WI, USA) was used to assess the relative rate of cell proliferation by measurement of ATP content present in cells according to instructions provided by the manufacturer. Briefly, cells were washed three times in HBSS and resuspended in experiment media as previously described. A total of 1 × 10^4^ cells were seeded in 100 μL medium per well in a 96-well optical plate. Experiments were run at 37°C in 5% CO_2_. After 24 h, the provided assay reagent was added to the wells, after which the plate was agitated on a microplate shaker for 2 minutes. The plates were subsequently kept at room temperature for 10 minutes before luminescence was quantified. The luminescent signal was recorded with a Victor3 plate reader and Wallac 1420 Workstation software (PerkinElmer Inc.).

### Immunoblotting

Cells were treated as indicated, collected, and lysed. Immunoblotting method was performed as previously described [48]. Images were acquired using LI-COR Odyssey Fc (LI-COR, Lincoln, Nebraska) and analyzed with Image Studio Software (LI-COR).

### Nanostring analysis

Primary cells from patients with MM (*n* = 25) and MGUS (*n* = 3), and PBMCs from healthy donors (*n* = 2) were prepared as previously described. For mRNA transcript counting the nCounter Human Custom Kit (SQ-18078, Nanostring Technologies, Seattle, WA, USA) and nCounter Technology (Nanostring Technologies) was used following the manufacturer’s protocol. The experiment was performed using the nCounter Analysis System, consisting of the nCounter Prepstation and nCounter Digital Analyzer. The standard mRNA Gene-expression experiment protocol provided by Nanostring was used. Briefly, 100 ng total RNA from patient samples and human myeloma cell lines (*n* = 8) was hybridized with reporter probes overnight at 65°C. Calculations of transcript numbers were done by the nSolver Analysis Software (Nanostring Technologies). Sample data was normalized against internal kit positive controls and housekeeping genes.

### Gene expression data

RNA sequencing data from CD138^+^ plasma cell bone marrow samples from 771 newly diagnosed MM patients were downloaded from the IA14 release of the CoMMpass trial database (https://research.themmrf.org). Data on overall survival (OS) and progression-free survival (PFS) were available for 767 patients. For survival analyses, patient samples taken at diagnosis were separated into *ST14* high-expressing (upper 10th percentile, *n* = 75) and non-expressing patients (Transcripts Per Million (TPM)<1.0, *n* = 281). We also analyzed *ST14*-expression in 37 patients at diagnosis and the time of last progression based on RNA-sequenced CD138^+^ cells from longitudinal samples. Survival and prognostic analyses were performed in GraphPad Prism 8.0 (GraphPad Software, USA). “Time” and “Status/Censoring” was acquired from the clinical data in CoMMpass. Survival curves were plotted using the Kaplan-Meier method.

### Statistics

The statistical differences of the patient data were determined using either log-rank test or Wilcoxon signed-rank test, as indicated. Student’s *t*-test was used for comparisons between two groups. All statistical analyses were performed with GraphPad Prism 8.0 (GraphPad Software, USA).

## SUPPLEMENTARY MATERIALS



## Abbreviations

BSA: Bovine serum albumin
CXCR4: CXC chemokine receptor 4
FCS: Fetal calf serum
HAI-1: Hepatocyte growth factor activator inhibitor 1
HAI-2: Hepatocyte growth factor activator inhibitor 2
HBSS: Hanks’ balanced salt solution
HGF: Hepatocyte growth factor
IL-6: Interleukin-6
MGUS: Monoclonal gammopathy of undetermined significance
MM: Multiple myeloma
MSP-1: Macrophage-stimulating protein 1
PBMC: Peripheral blood mononuclear cell
SDF-1α: Stromal cell-derived factor-1 alpha
SFK: Src family kinases
uPA: Urokinase-type plasminogen activator
