# Supplementary material for: The serine protease matriptase inhibits migration and proliferation in multiple myeloma cells
Source: Oncotarget. 2022 Oct 20;13:1175–86. doi: 10.18632/oncotarget.28300 (PMC9584456; doi:10.18632/oncotarget.28300)
Supplement: Supplementary file 1 [file oncotarget-13-28300-s001.pdf]

The serine protease matriptase inhibits migration and proliferation in multiple myeloma cells

SUPPLEMENTARY MATERIALS

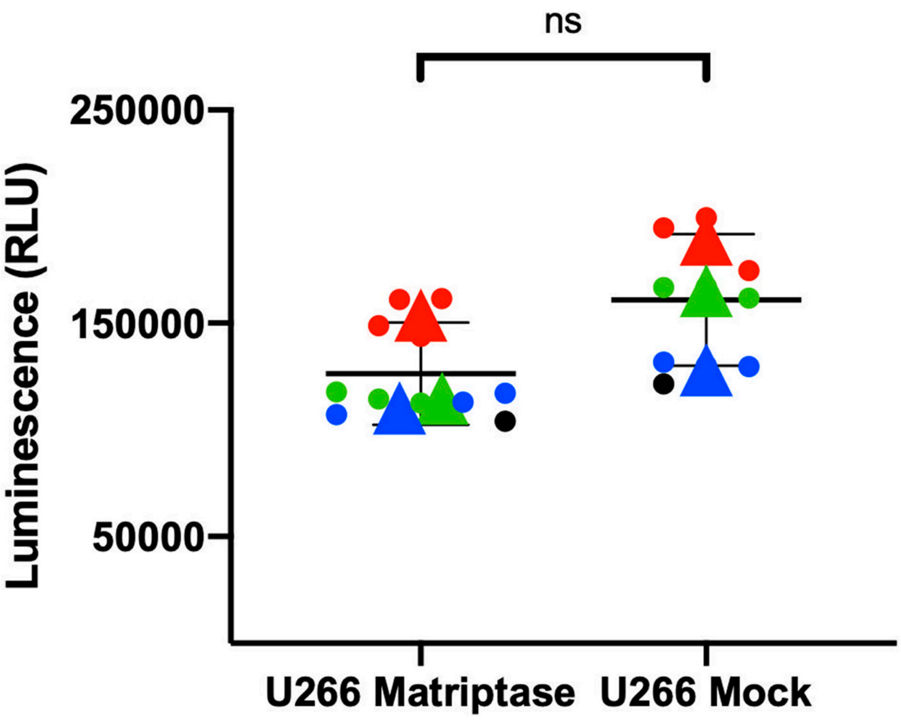

Supplementary Figure 1: Proliferation in U266 matriptase overexpression and control cells. Cell proliferation was measured by the CellTiter-Glo assay. The mean (±SD) of three independent experiments is shown. Each dot represents one technical replicate and dots in the same color correspond to one of the biological replicates. Triangles represent the mean of each biological replicate. *p*-value was calculated by unpaired Student's *t*-test based on the average from each independent experiment. Abbreviation: ns: not significant. ($p > 0.05$).

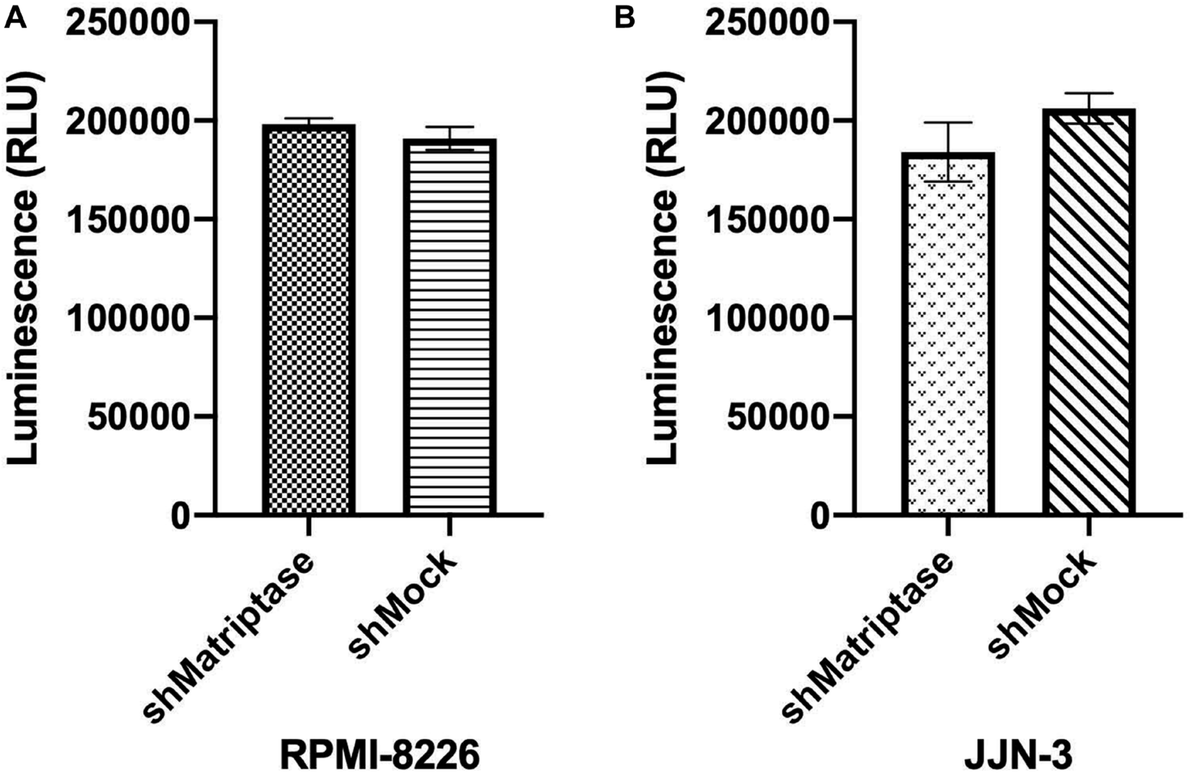

Supplementary Figure 2: Proliferation in matriptase knockdown and control cells. Cell proliferation was measured in (**A**) RPMI-8226 and (**B**) JJN-3 matriptase knockdown (shMatriptase) and control (shMock) cells by the CellTiter-Glo assay. Each bar represents the mean (±SD) of at least three technical replicates. One representative of at least three independent experiments is shown.

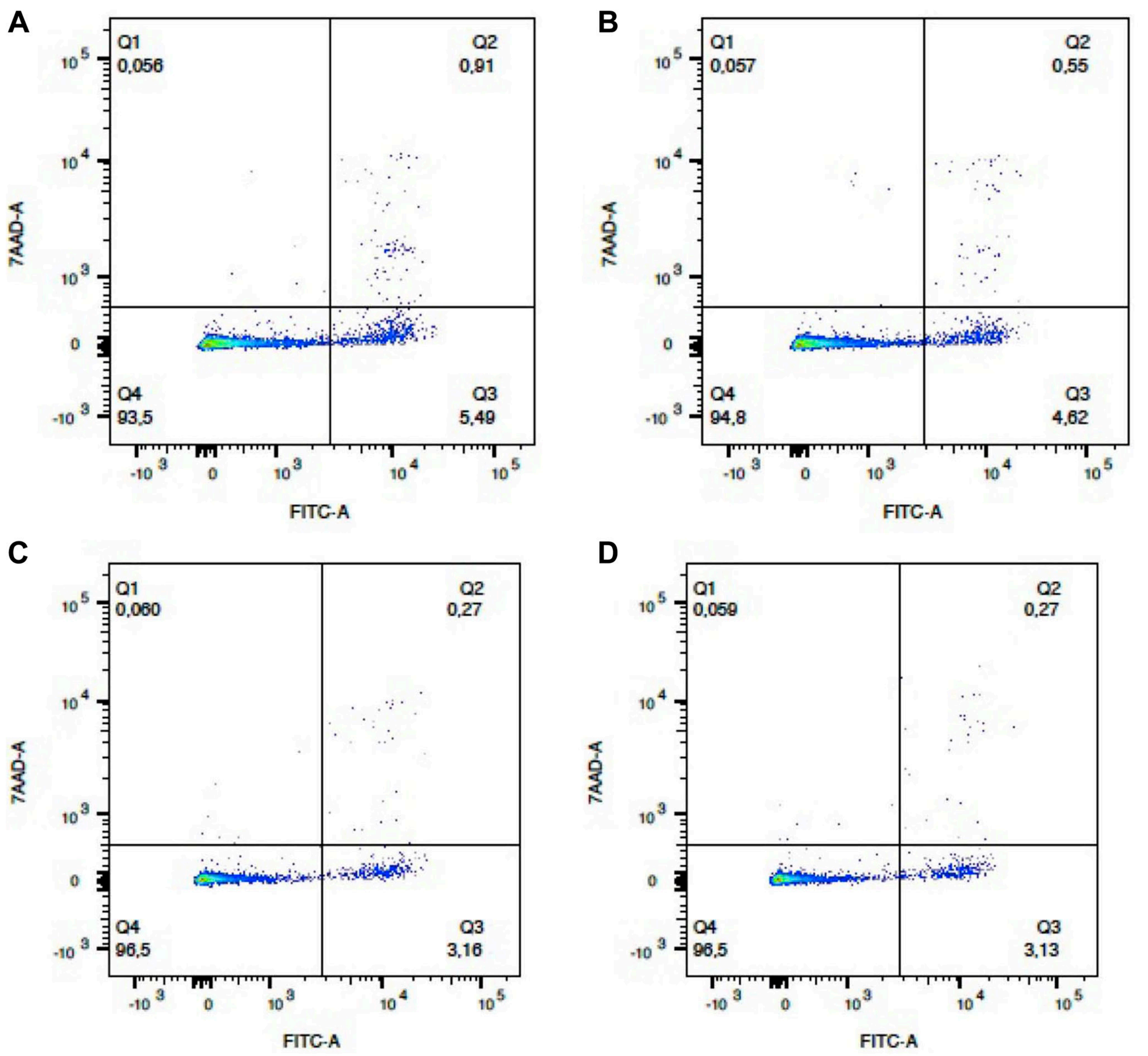

Supplementary Figure 3: Cell viability in INA-6 matriptase overexpression and control cells. INA-6 (**A**, **B**) Matriptase and (**C**, **D**) Mock cells were incubated in experimental media overnight before staining with annexin V-PI. The experiment was performed in duplicates, with each scatter plot representing one out of two technical replicates. Flow cytometry analysis was performed with LSR II (BD Biosciences) with FACS Diva Software (BD Biosciences). Samples were analyzed with FlowJo 10.4 (TreeStar, Ashland, OR, USA).

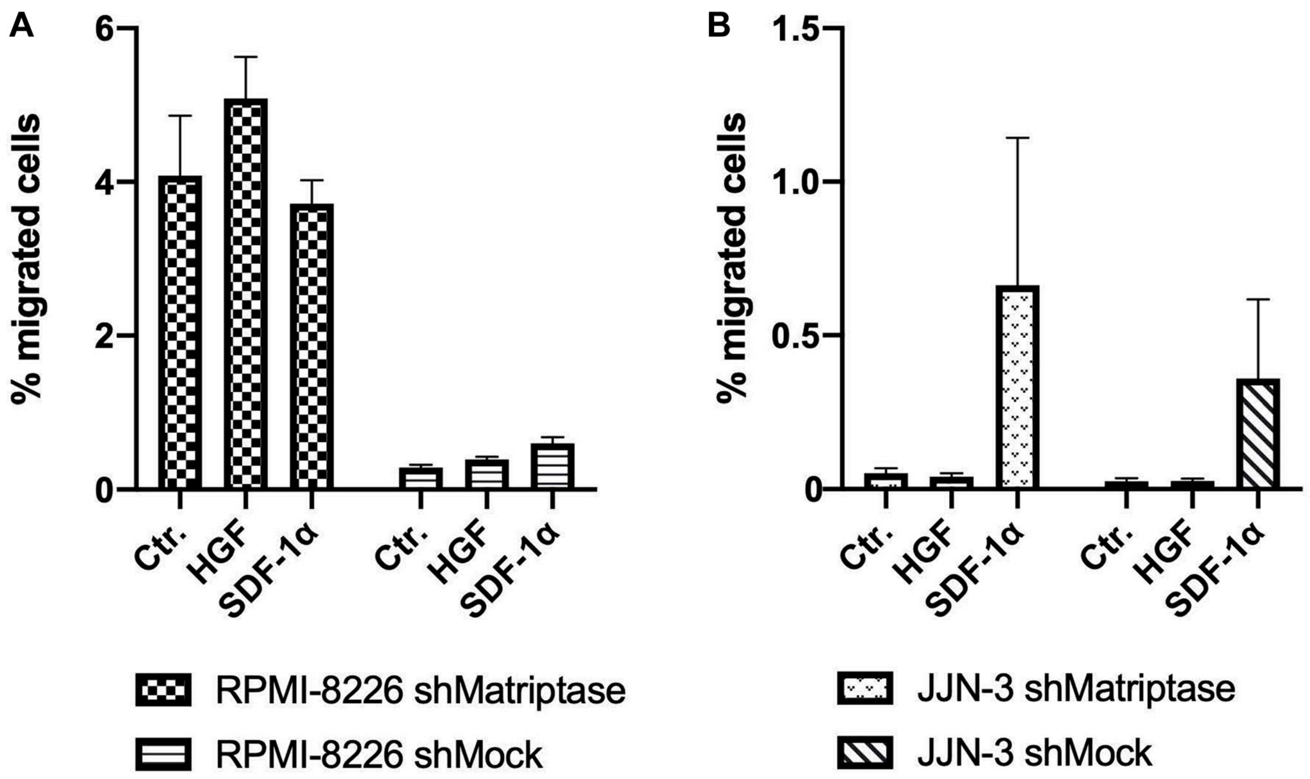

Supplementary Figure 4: Migration in matriptase knockdown cell lines upon cytokine stimulation. Migration of matriptase knockdown (shMatriptase) and control cells (shMock) in (**A**) RPMI-8226 and (**B**) JJN-3, respectively. Cells were seeded in the upper well of a two-chamber transwell migration assay. The promigratory cytokines HGF (150 ng/mL) or SDF-1α (75 ng/mL) were added to the lower wells as indicated. After 24 h incubation, cells in the bottom wells were counted and the percentage of migrated cells calculated. Bars represent the mean (±SD) of three repeated counts in two independent measurements. One representative of three independent experiments is shown in both figures.

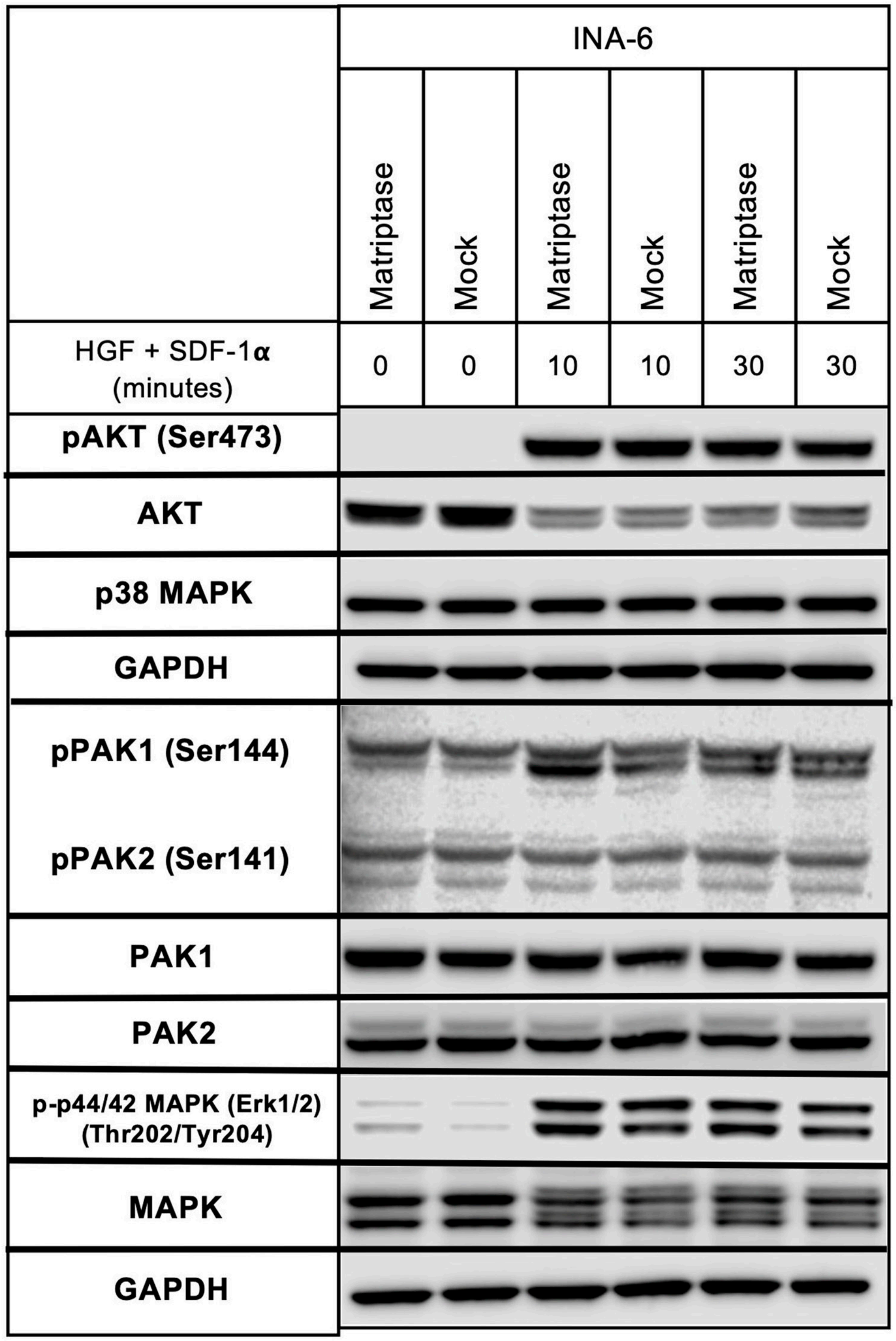

Supplementary Figure 5: Investigation of signaling pathways in INA-6 matriptase overexpression and control cells. INA-6 Matriptase and Mock cells were starved for 6 h in serum-free medium. Subsequently, cells were stimulated with HGF and SDF-1α for 10 and 30 min and probed with antibodies as indicated. Concentrations of HGF and SDF-1α was 150 ng/mL and 75 ng/mL, respectively. One representative blot out of three independent experiments is shown.

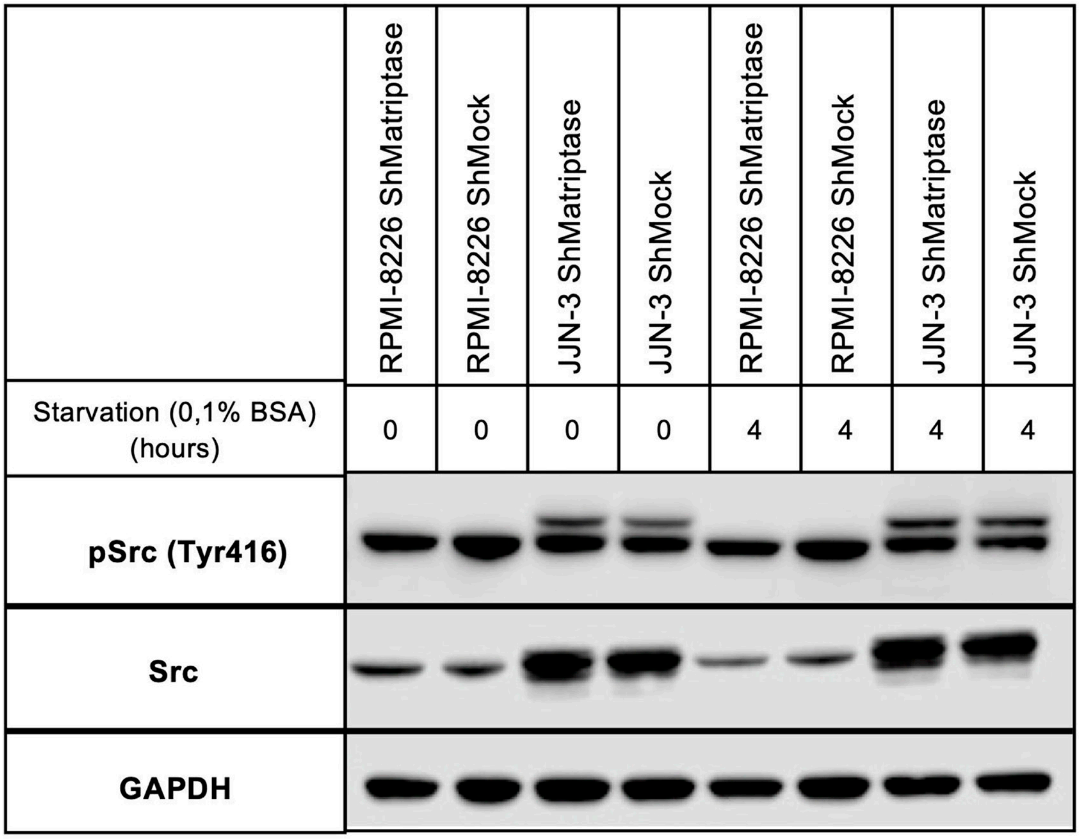

Supplementary Figure 6: Src activation in matriptase knockdown cell lines. Phosphorylated and total Src expression in matriptase knockdown (shMatriptase) and control cells (shMock) in RPMI-8226 and JJN-3, respectively. Cells were washed with HBSS and either harvested directly (0 h) or starved 4 h in serum-free medium before harvesting and probed with antibodies as indicated. One representative blot out of three independent experiments is shown.

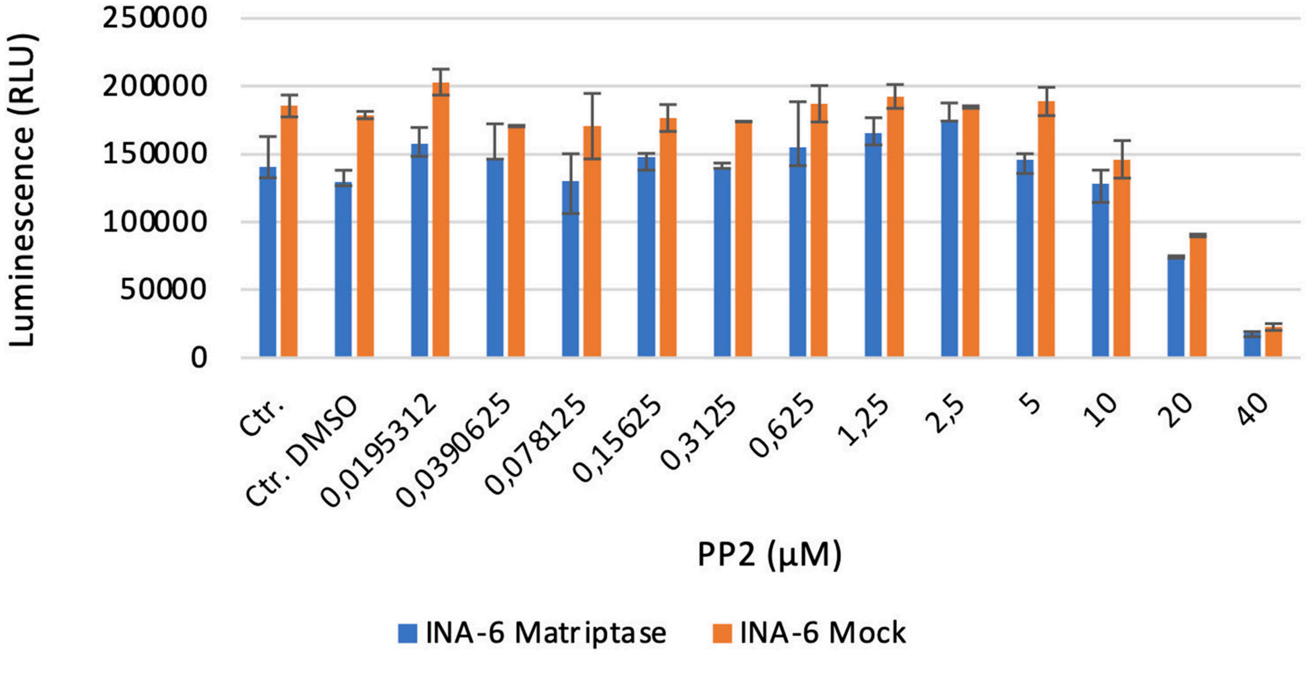

Supplementary Figure 7: Proliferation in INA-6 matriptase overexpression and control cells following PP2 Src inhibition. Cell proliferation was measured in INA-6 Matriptase and Mock cells by the CellTiter-Glo Assay. PP2 Src inhibitor was added in concentrations as indicated. One representative of at least two independent experiments is shown.

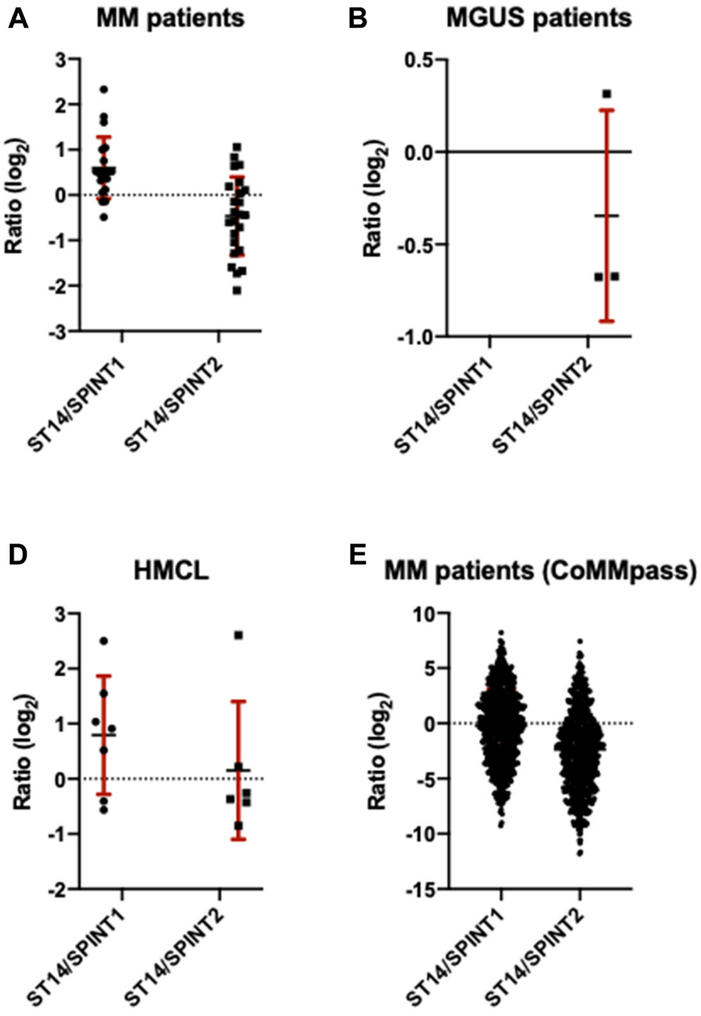

Supplementary Figure 8: *ST14/SPINT1* and *ST14/SPINT2* ratio in multiple myeloma and MGUS patients, healthy controls, and myeloma cell lines. *ST14/SPINT1* and *ST14/SPINT2* ratio in (**A**) primary cells from patients with multiple myeloma (MM, *n* = 25) and (**B**) monoclonal gammopathy of undetermined significance (MGUS, *n* = 3), (**C**) peripheral blood mononuclear cells (PBMCs, *n* = 2), (**D**) human myeloma cell lines (HMCLs, *n* = 8) and (**E**) MM patient samples from the MMRF CoMMpass IA14 release (*n* = 771). Ratios in A–D were based on the number of counts obtained from Nanostring mRNA expression analysis.

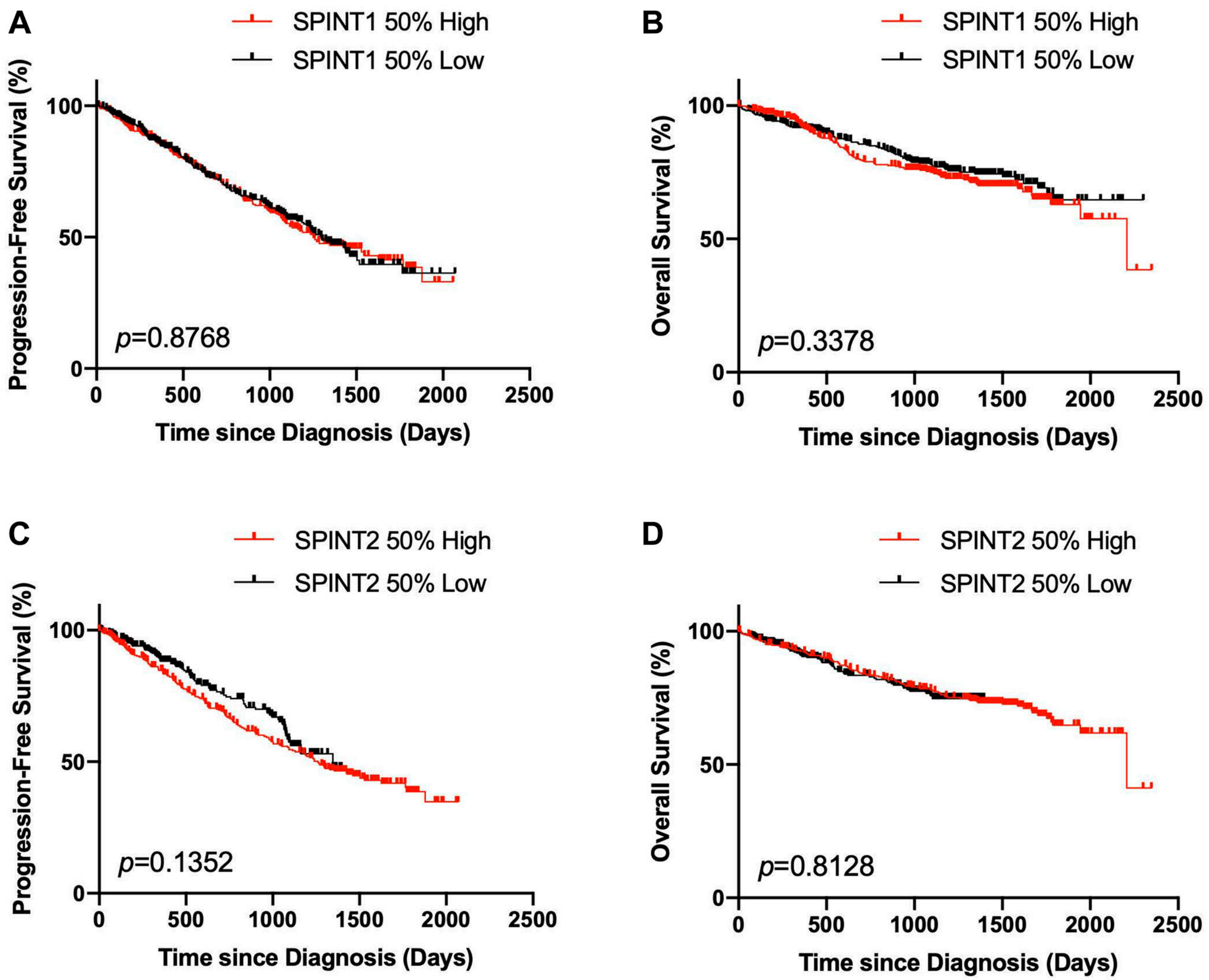

Supplementary Figure 9: Clinical relevance of HAI-1 and HAI-2 gene expression. Kaplan-Meier analysis with log-rank test for progression-free and overall survival data from CoMMpass IA14 stratified into cases above and below median for (**A**, **B**) HAI-1 and (**C**, **D**) HAI-2. A total of n = 767 cases were available for survival analysis of HAI-1 gene expression, while the equivalent number was n = 656 cases for HAI-2.

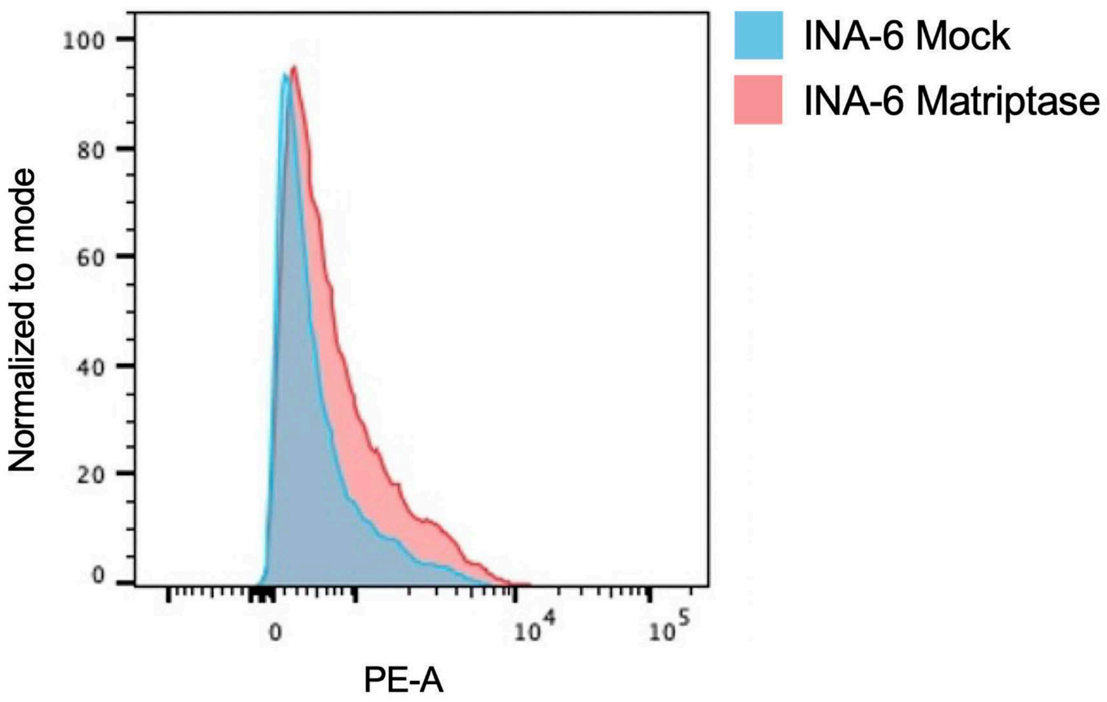

Supplementary Figure 10: CXCR4 expression on INA-6 matriptase and mock. Cell surface expression of CXCR4 was investigated in INA-6 Matriptase and Mock cells by flow cytometry. One replicate out of two representative is shown.

Supplementary Table 1: Available clinical data on MM and MGUS patients included in the nanostring analysis

		Gene expression					M-component		Cytogenetics	Staging	
**Diagnosis**	**Patient no.**	**ST14**	**SPINT1**	**SPINT2**	**Time of sample collection**	**Age**	**Serum M-component**	**Type**	**FISH**	**R-ISS at diagnosis**	**ISS at diagnosis**
MM	1	44770	0	10332	Unknown	68	IgG	Kappa	Normal	Unknown	II*
	2	19429	364	12669	Unknown	73	Unknown	Unknown	Unknown	Unknown	Unknown
	3	16060	75	2369	Relapse	69	IgG	Lambda	Normal	I	I
	4	6132	153	5636	Unknown	63	IgA	Kappa	Not analyzed	Unknown	II*
	5	5096	2146	7421	Diagnosis	75	IgG	Kappa	Unsuitable for analysis	Unknown	III
	6	2997	920	2331	Unknown	47	IgG	Lambda	Normal	Unknown	Unknown
	7	2719	0	7314	Diagnosis	66	IgG	Kappa	Normal	I	I
	8	1229	542	4650	Diagnosis	69	IgA	Lambda	Normal	II	II
	9	983	323	514	Unknown	84	FLC	Unknown	t(4;14)	Unknown	Unknown
	10	612	466	1440	Relapse	51	IgG	Lambda	Normal	Unknown	Unknown
	11	503	151	9603	Unknown	77	IgA	Lambda	Not analyzed	Unknown	II*
	12	436	40	38	Unknown	74	FLC lambda	Lambda	Normal	Unknown	II*
	13	356	108	1418	Unknown	67	IgG	Kappa	t(4;14)	Unknown	Unknown
	14	352	101	2516	Diagnosis	59	IgA	Kappa	t(4;14)	II	II
	15	348	310	5728	Unknown	70	Unknown	Unknown	Unknown	Unknown	Unknown
	16	246	25	1262	Unknown	82	Unknown	Unknown	Unknown	Unknown	Unknown
	17	206	0	45	Unknown	49	Ig	Lambda	Normal	Unknown	III*
	18	175	52	1937	Unknown	66	IgG	Kappa	Normal	Unknown	III*
	19	140	196	5434	Relapse	48	IgA	Lambda	Normal	Unknown	Unknown
	20	95	133	5091	Diagnosis	77	IgG	Kappa	t(4;14)	Unknown	Unknown
	21	62	190	2904	Diagnosis	72	IgG	Lambda	del17p	III	III
	22	58	10	82	Relapse	80	IgA	Kappa	del13q	II	II
	23	53	26	6770	Diagnosis	66	IgG	Kappa	Normal	II	II
	24	31	0	86	Relapse	75	FLC lambda	Lambda	t(4;14)	Unknown	Unknown
	25	0	0	4869	Diagnosis	58	IgG	Unknown	Normal	Unknown	I*
MGUS	1	19442	0	9426	Diagnosis	62	IgG	Kappa	Not analyzed	Unknown	Unknown
	2	3327	0	15803	Diagnosis	71	IgG	Lambda	Normal	Unknown	Unknown
	3	2546	0	12040	Diagnosis	27	FLC lambda	Lambda	Unsuitable for analysis	Unknown	Unknown

*Not at diagnosis.
